# Liquid First Is “Solid” in Naïve Non-Small Cell Lung Cancer Patients: Faster Turnaround Time With High Concordance to Solid Next-Generation Sequencing

**DOI:** 10.3389/fonc.2022.912801

**Published:** 2022-06-15

**Authors:** Or Sehayek, Waleed Kian, Amir Onn, Ronen Stoff, Hadas Gantz Sorotsky, Melanie Zemel, Jair Bar, Yulia Dudnik, Hovav Nechushtan, Yakir Rottenberg, Lior Soussan-Gutman, Addie Dvir, Laila C. Roisman, Nir Peled

**Affiliations:** ^1^ Ben-Gurion University, Be’er Sheva, Israel; ^2^ The Institute of Oncology, Shaare Zedek Medical Center, Jerusalem, Israel; ^3^ Sheba Medical Center, Ramat Gan, Israel, and Tel Aviv University Medical School, Tel Aviv, Israel; ^4^ Soroka Medical Center, Ben-Gurion University, Be’er Sheva, Israel; ^5^ Department of Oncology, Hadassah Medical Organization and Faculty of Medicine, Hebrew University of Jerusalem, Jerusalem, Israel; ^6^ Rhenium Oncotest Ltd., Modi’in, Israel

**Keywords:** circulating tumor DNA (ctDNA), turnaround time (TAT), driver mutation, liquid biopsy, non-small cell lung carcinoma (NSCLC)

## Abstract

**Purpose:**

Molecular profiling is crucial in naïve non-small cell lung cancer (NSCLC). While tissue-based analysis is challenged by turnaround time and scarcity of tissue, there is increasing demand for liquid biopsy. We aimed to analyze the use of upfront liquid biopsy as a molecular profiling approach.

**Methods:**

This retrospective multicenter, non-interventional study compared findings and turnaround times of liquid vs. standard-of-care (SOC) tissue-biopsy molecular profiling. The study included naïve advanced NSCLC patients with available liquid biopsy (Guardant360 CDx).

**Results:**

A total of 42 consecutive patients (60% men; median age, 69.5 [39–87] years; 86% stage IV NSCLC) were identified between September 2017 and December 2020. Liquid-biopsy analysis provided results for all 42 patients, whereas the tissue-based analysis failed in 5 (12%) patients due to insufficient tumor samples. In 17 patients, 18 actionable driver mutations were identified. Eleven mutations were detected by both approaches (i.e., concordance of 61%), 4 only by liquid biopsy and 3 only by tissue biopsy. The median time from the molecular request to receiving the molecular solid report on the last biomarker was 21 (range: 5–66) days, whereas the median time from blood draw to the liquid-biopsy results was 10.5 (7–19) days. The median time between the availability of liquid-biopsy findings and that of the last biomarker was 5 days. Treatment changes following the liquid-biopsy results were observed in 3 (7%) patients.

**Conclusion:**

Performing liquid-biopsy upfront is feasible and accurate and allows a shorter time for treatment in NSCLC, especially when tumor tissue is scarce.

## Introduction

The treatment journey in non-small cell lung cancer (NSCLC) depends on the driver existence in both early and advanced diseases. Treatment relies on the detection of driver mutations in the *epidermal growth factor receptor* (*EGFR*), *c-ros oncogene 1* (*ROS1*), *anaplastic lymphoma kinase* (*ALK*), *BRAF*, *MET exon 14 skipping* mutations, *KRAS G12C*, *RET* rearrangements, and *NTRK* gene fusions. In addition, other targets are emerging, including *HER2* mutations, *EGFR exon 20* insertions, and others such as *NRG1* fusions (1). These therapies are increasingly prevalent in the treatment of NSCLC, and their use is associated with significantly improved overall survival (2–6), while immune checkpoint inhibitors (ICIs) have been shown to be more effective in non-oncogene-addicted patients (7).

Currently, diagnosis and molecular profiling used for guiding treatment decisions in advanced NSCLC are typically performed on tissue obtained in an invasive biopsy procedure (6, 8–10). Liquid biopsies enable the diagnosis and molecular profiling of NSCLC through analysis of circulating tumor cells (CTC) and tumor cell-free DNA (cfDNA) using next-generation sequencing (NGS) to simultaneously assess multiple cancer-specific mutations. Unlike traditional tissue biopsy, liquid biopsy is minimally invasive and provides dynamic and accessible genomic profiling. Liquid biopsy is particularly beneficial when tumor tissue is scarce or unavailable and can also be used to track disease progression, therapy response, and the emergence of resistance (6, 11, 12).

The recent consensus statement from the International Association for the Study of Lung Cancer (IASLC) states that in newly diagnosed NSCLC patients with tumor tissue available for initial genotyping, liquid biopsy could be used if tissue testing proves inadequate. The statement also points out that a concurrent approach seems a practical option for patients with small tumor biopsies with uncertain adequacy for tumor genotyping, although the concurrent approach is associated with high cost (12).

One of the commercially available tools for liquid biopsy is Guardant360^®^ CDx (Guardant Health, Redwood City, CA, USA). This is an NGS-based cfDNA test that assesses single-nucleotide variants (SNVs) in over 70 genes, as well as insertion–deletion (indel), fusion alterations, and copy-number amplifications (in select genes) (5, 13).

In this retrospective study, we compared the molecular profiling findings and turnaround times between upfront liquid-based and the standard-of-care (SOC) tissue-based approaches in a real-life setting.

## Methods

### Study Design and Patients

**Figure 1:**
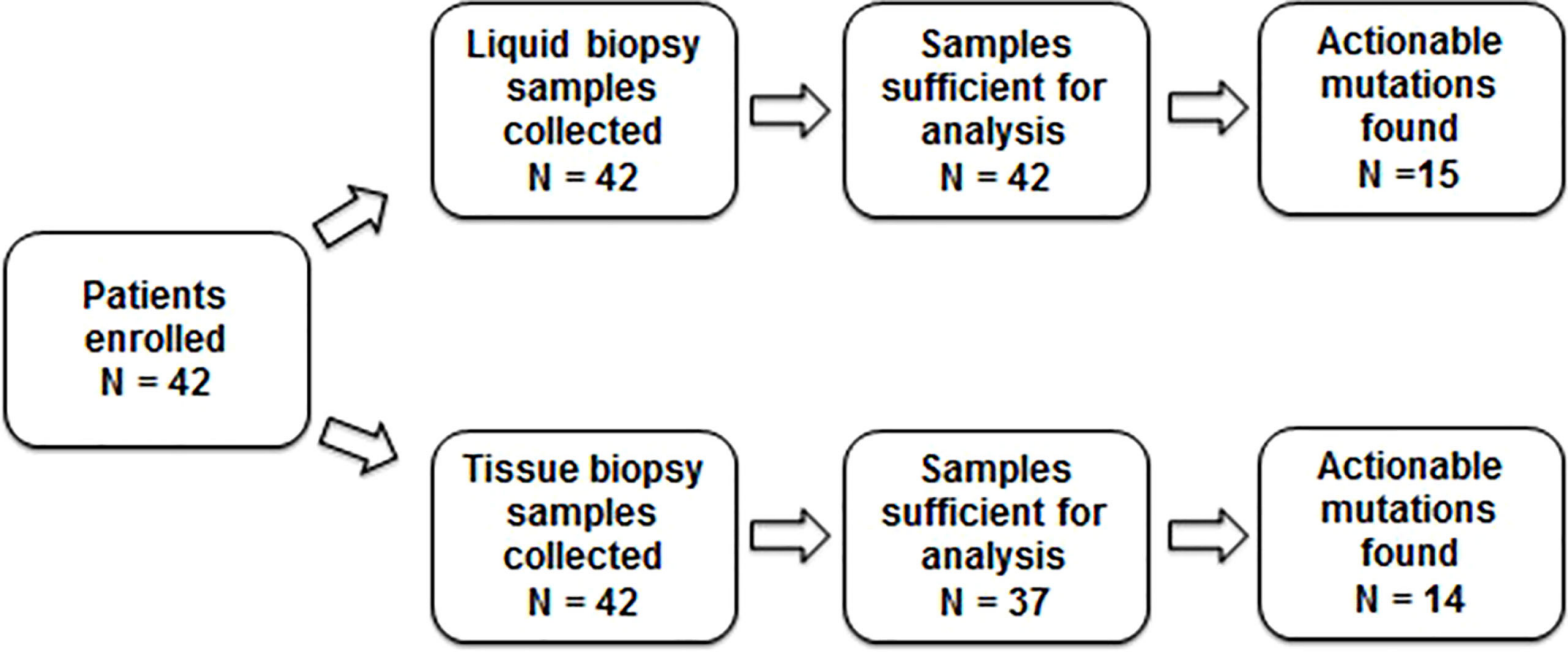
Study design.

This retrospective multicenter cohort study included patients with biopsy-proven advanced NSCLC who were diagnosed between September 2017 and December 2020, were treatment naïve, were candidates for systemic therapy, and for whom the treating physician ordered both standard tissue-based genotyping test and NGS-based liquid biopsy (Guardant360 CDx) on the same day (**Figure 1**).

The study was approved by the institutional review boards of the participating centers and was conducted in accordance with the Declaration of Helsinki. The study was granted a waiver for obtaining patient consent.

### Molecular Profiling

SOC tissue genotyping was performed on biopsy material in each of the participating centers according to the standard practices in each center. Tissue genotyping included analysis of *EGFR* mutations with real-time PCR and narrow-spectrum NGS assays. *ALK* rearrangements were assessed with immunohistochemistry, fluorescence *in situ* hybridization (FISH), or both. The presence of *MET* and *RET* mutations was assessed using NGS. *ROS1* mutations were detected with FISH or immunohistochemistry. NGS-based liquid biopsy was performed on blood samples using Guardant360 CDx.

Turnaround time was calculated for both liquid and tissue biopsies from the date the assays were requested by the referring physician to the date the results were received. For the tissue biopsies, if not all the results were received on the same day, the turnaround time was defined until the date the last result became available.

### Statistical Analysis

Descriptive statistics were used to summarize patient characteristics and molecular profiling findings. Concordance between the liquid and tissue biopsies was calculated by determining the number of cases of genomic alterations that were found in both biopsies out of the total cases of genomic alterations identified.

## Results

### Patient Characteristics

The final analysis included 42 patients. The majority were male (60%), the median age was 69.5 (range, 39–87) years, 29% were never smokers, and 86% had stage IV disease at diagnosis. Overall, 86% of the patients had adenocarcinoma, 8% had bone metastasis at diagnosis, and 26% had metastases in ≥3 sites. In total, 48% of the patients underwent endobronchial ultrasound (EBUS) or transbronchial biopsy. The tumor content was >20% in 43% of the samples. Programmed death-ligand 1 (PD-L1) status was >50% in 26% of the patients (**Table 1**).

**Table 1:** Baseline patient and tumor characteristics.

Characteristics	N = 42
**Age**	
Median (range), years	69.5 (39–87)
**Gender, n (%)**	
Male	25 (60)
Female	17 (40)
**Smoking history, n (%)**	
Former	19 (45)
Never	12 (29)
Current	10 (24)
Unknown	1 (2)
**Histology, n (%)**	
Adenocarcinoma	36 (86)
Squamous cell carcinoma	3 (7)
Mixed (adenocarcinoma and squamous cell carcinoma)	2 (5)
Large-cell carcinoma	1 (2)
**Stage of disease at diagnosis, n (%)**	
III	6 (14)
IV	36 (86)
**Sites of metastasis at diagnosis, n (%)**	
Bone	16 (38)
Brain	7 (16)
Liver	6 (14)
≥3 sites	11 (26)
**Type of biopsy, n (%)**	
EBUS or trans bronchial	20 (48)
Trans-thoracic core	11 (26)
Pleural effusion	6 (14)
Surgical	4 (10)
Other	1 (2)
**Tumor content in biopsy, n (%)**	
<5%	5 (12)
5%–20%	16 (38)
>20%	18 (43)
N/A	3 (7)
**PD-L1 status, n (%)**	
>50%	11 (26)
1%–49%	13 (31)
<1%	13 (31)
N/A	5 (12)

EBUS, endobronchial ultrasound; N/A, not available/not applicable; PD-L1, programmed death-ligand 1.

### Molecular Profiling

The molecular profiling results were analyzed in the context of the 2020 National Comprehensive Cancer Network^®^ (NCCN^®^) guidelines for NSCLC (14) and are summarized in **Table 2**. The Guardant360 test provided full NGS molecular analysis results for all 42 patients; however, tissue molecular analysis was not performed for 5 patients (12%) due to insufficient tumor samples. Overall, in 17 patients, 18 actionable driver mutations were identified (2 genomic alterations were identified in the same patient). Fifteen mutations were detected by liquid biopsy and 14 by tissue biopsy (i.e., 11 mutations were detected by both approaches, 4 only by liquid biopsy, and 3 only by tissue biopsy). Thus, of the 18 genomic alterations identified, 11 (61%) cases were concordant, and 7 (39%) cases were discordant.

**Table 2:** Molecular profiling results.

Driver mutations, n (%)	Tissue biopsy N = 37^1^	Liquid biopsy N = 42
*ALK* rearrangement	2 (5)^2^	1 (2)^2^
*EGFR*	11 (30)^2^	9 (21)^2^
ROS1	1 (3)^3^	2 (4)^3^
MET exon 14 skip	0^3^	2 (4)^4^
*RET*	0	1 (2)
Genomic alterations (according to NCCN 2020)	14 (38)	15 (36)
Non-actionable	22 (59)^5^	25 (59)^5^
Total mutations	36 (97)^6^	40 (95)^6^

ALK, anaplastic lymphoma kinase; EGFR, epidermal growth factor receptor; NCCN, National Comprehensive Cancer Network; ROS1, c-ros oncogene 1.

^1^ For 5 patients (12%), the molecular analysis could not be performed due to insufficient tumor samples.

^2^ One patient had an EGFR mutation (identified by tissue and not a liquid biopsy) and an ALK rearrangement (identified both in the tissue and liquid biopsies).

^3^ In one case, ROS1 mutation was identified in liquid biopsy, whereas in the corresponding tissue sample, the analysis could not be performed due to insufficient tissue samples.

^4^ In one case, MET exon 4 skip was identified in liquid biopsy, whereas in the corresponding tissue sample, the analysis could not be performed due to insufficient tissue sample.

^5^ In three cases, non-genomic alterations were identified in liquid biopsy, whereas in the corresponding tissue samples, the analyses could not be performed due to insufficient tissue samples.

^6^ Overall, in 4 cases, mutations were identified in the liquid biopsy, whereas in the corresponding tissue samples, analyses could not be performed due to insufficient tumor samples.

Specifically, in the tissue-based analysis, 11 cases with *EGFR* mutations and 2 with *ALK* rearrangements were identified. The liquid-based analysis missed 2 cases with *EGFR* mutations and one with an *ALK* rearrangement. The liquid-based analysis identified 2 *ROS1* mutations, whereas the tissue-based analysis detected only one (in the other case, an insufficient tumor sample prohibited the analysis). Liquid biopsy detected *MET*-ex14 skip mutations in 2 cases, whereas tissue biopsy detected none (in one of these cases, the analysis was not performed due to an insufficient tumor sample). Also, liquid biopsy but not tissue biopsy detected one case of *RET* fusion.

### Turnaround Times

**Figure 2:**
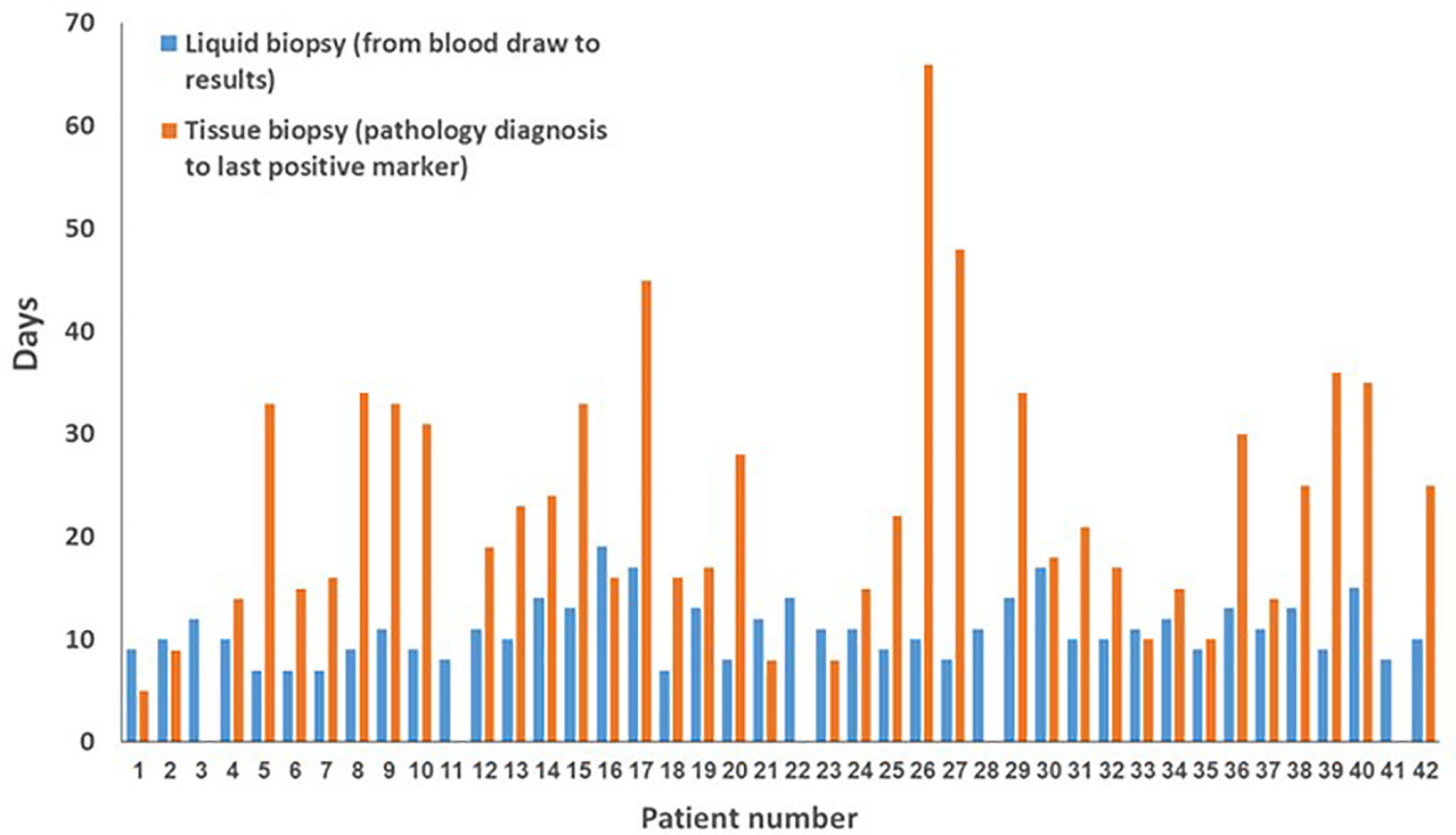
Turnaround times for liquid and tissue biopsy by patient.

Analysis of the turnaround times for the tissue-based and liquid-based analyses are described for each patient in **Figure 2** and summarized in **Table 3**. The median time from the histological diagnosis to receiving the tissue report on the last biomarker was 21 (range, 5–66) days, whereas the median time from blood draw to the cfDNA results was 10.5 (range, 7–19) days. The median time between the availability of cfDNA findings and that of the last biomarker result was 5 days.

**Table 3:** Turnaround times.

Turnaround time, days	Median (range)
**Liquid biopsy**	
From histological diagnosis to a blood draw	6 (−25 to 48)
From blood draw to final liquid molecular reports	10.5 (7 to 19)
From histological diagnosis to final liquid molecular reports	18 (−14 to 58)
**Tissue biopsy**	
From biopsy date to final tissue molecular reports	31 (13 to 78)
From histological diagnosis to final tissue molecular reports	21 (5 to 66)

### Changes in Treatment Decisions

Treatment changes following the liquid-biopsy results were observed in 3 (7%) of the 42 study patients. In the first case, the planned treatment was pembrolizumab; however, as the liquid biopsy identified an *EGFR* mutation, treatment with afatinib was initiated. Notably, approximately a month after afatinib treatment initiation, the tissue biopsy result, which also identified an *EGFR* mutation, became available. In the second case, chemotherapy was planned, as tissue biopsy was not performed due to insufficient tumor samples. Since the liquid biopsy identified *MET* exon 14 skip, treatment with a MET inhibitor (crizotinib) was initiated. In the third case, no genomic alterations were found in the tissue-based analysis; however, as the liquid biopsy identified a *KIF5B–RET* fusion, the patient enrolled in a clinical trial investigating selpercatinib (LOXO-292).

## Discussion

In this study, we compared tissue-based and liquid-based molecular analyses with respect to results and turnaround times in patients with advanced NSCLC and found concordant profiling results and a shorter turnaround time with the liquid biopsy. Also, the Guardant360 CDx test provided comprehensive molecular profiling for all 42 patients, whereas tissue-based molecular analysis was incomplete for 12% of the patients due to insufficient tumor material.

Overall, the liquid biopsy was as effective as tissue-based profiling in identifying NCCN-recommended actionable alterations. However, it did miss 2 of 11 *EGFR* mutations. This could result from low tumor DNA shedding, which is strongly associated with the quantity and sites of metastases (11) and is a known limitation of liquid biopsy. Indeed, both *EGFR*-positive patients had only lymph node metastatic dispersion. Furthermore, the liquid biopsy missed one rearrangement. Interestingly, the patient in question had distant metastatic sites, so this false-negative result is not due to low DNA shedding. It is established, however, that the sensitivity of analyzing cfDNA for rearrangements is lower than that for SNVs or indels because the cfDNA is highly fragmented resulting in lower amounts of mappable sequence to detect the fusion. It is also estimated that *ALK* rearrangements are more easily detectable utilizing cfRNA than cfDNA techniques (15, 16).

The RET mutation (KIF5B–RET fusion), which is found in 1%–2% of lung cancer patients and is also targetable by multiple treatment modalities, was discovered in one patient by liquid biopsy but missed by tissue biopsy. Rich et al. stressed the benefits of employing plasma NGS platforms over SoC tissue testing for comprehensive tumor genotyping, particularly when referring to rare targets such as RET (17).

*MET* ex14 skip mutations were detected using liquid biopsy in 2 cases and by tissue biopsy in one case (in the other case, the tumor sample was insufficient). This finding is consistent with prior research indicating that this driver mutation, which is targetable by several therapeutic approaches, is found in approximately 3%–4% of lung adenocarcinomas. Notably, cMET skip14 mutation was missed by solid NGS in a recent Canadian study (VALUE), which examined clinical outcomes and utility of liquid biopsy in naïve stage IV lung adenocarcinoma patients. In this study involving 146 patients, 2 cases of cMET skip14 were identified by solid and liquid biopsies and 2 only by solid and not by liquid biopsy, and 7 were identified only by liquid biopsy. Thus, our results suggest that liquid biopsy is a reliable diagnostic technique for detecting genomic alterations in NSCLC.

Previous studies have shown similar shorter turnaround times with liquid compared to tissue-based biopsy in NSCLC and shorter time to treatment. These studies include the Canadian VALUE study where the mean turnaround time ( ± SD) was 7.7 ± 1.6 vs. 20.8 ± 9.8 days for liquid vs. tissue-based biopsy, as well as the North American NILE study involving 282 patients with advanced lung adenocarcinoma where the time to treatment was significantly shorter with liquid biopsy compared to tumor tissue molecular profiling (median 18 vs. 31 days, respectively, *p* = 0.0008) (18, 19). Our study is also consistent with the VALUE study with respect to the high concordance rate between the liquid and solid biopsy results (18).

Our findings regarding the shorter turnaround time with liquid biopsy have clinical implications for clinical practice. Shorter turnaround times are particularly relevant for frail patients for whom waiting for test results or undergoing additional biopsies if the quantity of the tumor sample is insufficient may be unfeasible.

Recent research compared the cost-effectiveness of NGS with single-gene testing techniques in patients with metastatic NSCLC from the standpoint of the Centers for Medicare & Medicaid Services (CMS) and US commercial payers. They discovered that using upfront NGS testing in patients with metastatic NSCLC (mNSCLC) resulted in significant cost savings and shorter time-to-test results for both CMS and commercial payers (20–22).

This pilot study is limited by the small number of patients and by its retrospective design. Also, as this was a retrospective study reflecting real-life clinical practices, the tissue biopsy analyses varied across the participating centers (with respect to the gene panels examined, whether NGS was performed, etc.). Another limitation is the lack of detailed records of the tumor load of each patient, which is known to correlate with the chance of success of liquid biopsy studies. Another limitation of our study is that we did not include data on variant allele frequency (VAF). Recent research, however, has found that VAF is related to tumor burden and corresponds with prognosis. Patients with reduced VAF, for example, reacted better to atezolizumab in the B-F1RST research (23). Furthermore, at very low VAF, some discordance between tissue and plasma NGS may be identified (24). Future studies with larger sample sizes and predefined/centralized tissue biopsy analyses are warranted.

In conclusion, considering the improved turnaround time and high concordance, performing liquid biopsy upfront appears to be an important NSCLC management strategy, especially when tumor tissue is scarce. Further studies are required to study “liquid first” or “liquid only” in NSCLC.

## Data Availability Statement

The original contributions presented in the study are included in the article/supplementary material. Further inquiries can be directed to the corresponding authors.

## Ethics Statement

The studies involving human participants were reviewed and approved by 0072-19-SOR. Written informed consent to participate in this study was provided by the participants’ legal guardian/next of kin.

## Author Contributions

OS: writing—original draft, visualization, and investigation. WK: writing—original draft, visualization, conceptualization, methodology, and review. AO: visualization and resources. RS: visualization and resources. HS: visualization and resources. MZ: editing. JB: visualization, resources, and review and editing. YD: visualization and resources. HN: visualization, resources, and review and editing. YR: visualization and resources. LS-G: visualization and resources. AD: visualization and resources. LR: writing—original draft, visualization, conceptualization, methodology, resources, and review and editing. NP: visualization, conceptualization, methodology, resources, and review and editing. All authors listed have made a substantial, direct, and intellectual contribution to the work and approved it for publication.

## Conflict of Interest

AD and LS-G are employed and are shareholders at Rhenium Oncotest Ltd. NP declares advisor and honorarium from and research with AstraZeneca, Bayer, Boehringer Ingelheim, Bristol-Myers Squibb, Eli Lilly, Foundation Medicine, Guardant360, Merck, MSD, Novartis, NovellusDx, Pfizer, Roche, and Takeda. YR declares a speakers bureau from MSD, AZD, Roche, Dexcel, Medison, and Novartis and is a consultant for MSD and Takeda. JB declares research funding (for the institute) and advisor fees from MSD, AstraZeneca, Pfizer, Takeda, AbbVie, Roche, Novartis, and Merck Serono and share options from Causalis.

The remaining authors declare that the research was conducted in the absence of any commercial or financial relationships that could be construed as a potential conflict of interest.

## Publisher’s Note

All claims expressed in this article are solely those of the authors and do not necessarily represent those of their affiliated organizations, or those of the publisher, the editors and the reviewers. Any product that may be evaluated in this article, or claim that may be made by its manufacturer, is not guaranteed or endorsed by the publisher.
